# Silicone Films Modified with Ethylene Glycol Dicyclopentenyl Ether Acrylate for Antimicrobial Silver Loading

**DOI:** 10.3390/polym17182482

**Published:** 2025-09-14

**Authors:** Orlando Padilla, Miguel S. Pérez-Garibay, Alejandro Camacho-Cruz, Emilio Bucio

**Affiliations:** 1 Departamento de Química de Radiaciones y Radioquímica, Instituto de Ciencias Nucleares, Universidad Nacional Autónoma de México, Ciudad Universitaria, Circuito Exterior, Mexico City 04510, Coyoacán, Mexico; miguel.s.garibay@gmail.com; 2 Facultad de Química, Universidad Autónoma de México, Ciudad Universitaria, México City 04510, Coyoacán, Mexico; cfqunam@yahoo.com.mx

**Keywords:** silicone, grafting, gamma radiation, EGDEA, silver, antimicrobial properties

## Abstract

In this research, silicone films (SR) were modified by grafting ethylene glycol dicyclopentenyl ether acrylate (EGDEA) through gamma-ray irradiation using both direct and pre-irradiation methods at a dose rate of 10.8 kGy/h, with doses ranging from 10 to 50 kGy. Several techniques, including TGA, DSC, contact angle measurement, mechanical testing, swelling, and FTIR, confirmed the grafting of EGDEA onto SR films. The highest grafting efficiency was achieved at 50 kGy using the direct method. Subsequently, SR-g-EGDEA films were loaded with silver for microbial testing, showing promising results for potential biomedical applications.

## 1. Introduction

Currently, a substantial increase in hospitalization cases has been reported, as well as the discovery of new diseases, leading to prolonged hospital stays and, in turn, generating opportunities for nosocomial infections [1]. In this sense, various medical devices used, such as intravenous catheters, heart valves, orthopedic implants, among others, are of interest to modify with antimicrobial agents since they coexist with various nosocomial pathogenic microorganisms [2].

SR is one of the polymeric materials with excellent biomedical applications, being used in several devices, since it does not present any reaction to blood flow and, in turn, presents both chemical and thermal stability, good mechanical properties, and biocompatibility [3,4]. On the other hand, SR, due to its hydrophobic nature, favors the adhesion of bacteria and proteins on its surface [5]. To overcome this, SR can be modified through the incorporation of monomers, allowing the capacity to load antibacterial agents and the improvement of wettability [6]

Grafting is a technique that allows the addition of covalently linked monomers to a polymer chain, enabling broader applications [7]. In this line, gamma-ray irradiation allows the modification of polymers by grafting, offering different methods such as direct and oxidative pre-irradiation methods [8].

**Figure 1:**
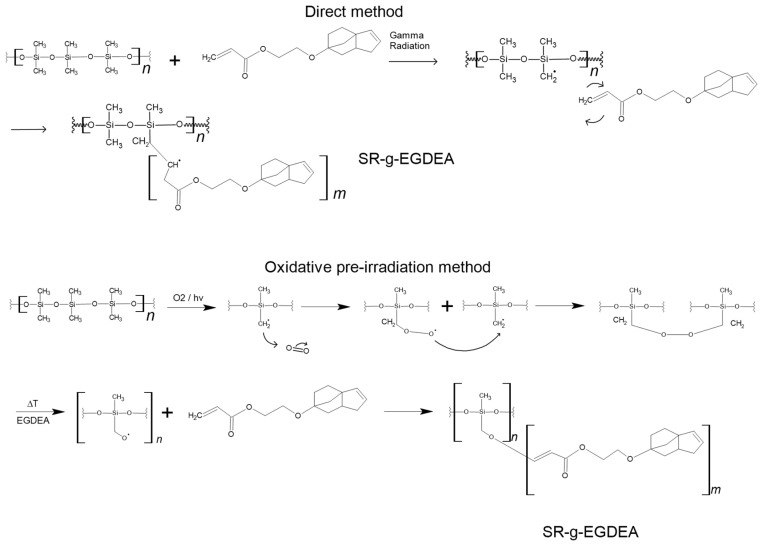
Mechanisms proposed for the grafting of EGDEA onto SR films.

In the direct method, the polymer is brought into contact with a mixture of monomer and solvent; radiation creates macroradical sites in the matrix that initiate graft polymerization and homopolymerization of the monomer [8]. The polymer is irradiated in the presence of air, which generates hydroperoxides or peroxides that decompose at high temperatures, then contacts the monomer to start the grafting reaction [9]. Both methods are illustrated in Figure 1.

Ethylene glycol dicyclopentenyl ether acrylate (EGDEA) is a substance that has recently gained interest due to its antibacterial properties [9,10,11].

Silver was probably the most essential antimicrobial before antibiotics were introduced in the 1940s, and is still used today in various medical applications because of its antibacterial effects and low toxicity [12]. Silver offers a broad spectrum option covering viruses, bacteria, and fungi [13,14]. To prevent bacterial resistance, silver can be combined with other polymeric materials to produce a synergistic effect [15,16].

Building on this, the research focused on modifying SR films by grafting EGDEA (SR-g-EGDEA) using gamma ionizing radiation, followed by loading them with antimicrobial silver, to obtain a material with enhanced wettability and antibacterial properties.

## 2. Materials and Methods

### 2.1. Materials

SR films with a density of 1.1 to 1.5 g/cm^3^ and 1 mm of thickness were acquired from Good-Fellow (Hunting, UK). Ethylene glycol dicyclopentyl ether acrylate and polyvinylpyrrolidone (360 KDa) were obtained from Sigma-Aldrich Co. (St. Louis, MO, USA). EGDEA was purified through reduced-pressure distillation. Hexanes were obtained from Meyer Chemical Reagents (86.18 g/mol) (Mexico City, Mexico). Petroleum ether, acetone, and ethanol were purchased from J.T. Baker (Mexico City, Mexico). Silver nitrate was purchased from DEQ Monterrey (Nuevo León, Mexico).

### 2.2. Synthesis of SR-g-EGDEA by the Direct Method

SR films were cut into 4 × 1 cm pieces and washed with ethanol for 2 h to remove impurities. Subsequently, the films were dried at 45 °C under vacuum for 4 h to determine their weight. Afterward, the films were placed into glass ampoules, and then 8 mL of 25% (*v*/*v*) EGDEA solution (Table 1) was added to each. Next, argon was pumped into the ampoules for 15 min, and they were then sealed to create an inert atmosphere. The ampoules were exposed to a 60Co γ-source (Gammabeam 651 PT, MDS Nordion, Ottawa, ON, Canada) at various radiation doses (10, 30, 50 kGy). Once irradiated, the films were extracted and washed with ethanol for 24 h to remove any homopolymer residues. The films were then dried under vacuum at 45 °C for 4 h for reweighing. The grafting percentage was calculated as follows.

(1) $$Grafting\;\left(\%\right)=\frac{\left(Wf-Wo\right)}{Wo}\times 100$$

where Wf = final weight of the grafted film and Wo = initial weight of the pristine film.

### 2.3. Synthesis of SR-g-EGDEA by the Oxidative Pre-Irradiation Method

SR films underwent the same initial washing and drying process as the direct method. Afterward, the films were placed into glass ampoules and exposed to a 60Co γ-source (Gammabeam 651 PT, MDSNordion, Ottawa, ON, Canada) at various radiation doses (10, 30, 50 kGy) in the presence of air. The procedure then continued as in the direct method: 8 mL of a 25% (*v*/*v*) EGDEA solution (Table 2) was added to each ampoule, which was then pumped with Ar and sealed. The sealed ampoules were incubated in a water bath at 60 °C and 80 °C for 72 h each. Finally, the films were extracted, washed, and dried following the same steps as the direct method. The grafting percentage was determined using Equation (1).

### 2.4. Fourier Transform Infrared Spectroscopy with Attenuated Total Reflectance (FTIR-ATR)

Films of SR, SR-g-EGDEA, and EGDEA homopolymer were dried for 12 h at 60 °C. Afterwards, films were analyzed using a Perkin-Elmer Spectrum 100 Spectrophotometer with a diamond tip from Perkin Elmer Cetus Instruments, Norwalk, CT, USA, performing 16 scans for each sample with the ATR mode.

### 2.5. Thermogravimetric Analysis (TGA)

Approximately 5–30 mg of each film of SR, SR-g-EGDEA, and EGDEA homopolymer were dried for 24 h at 60 °C. Then, the samples were placed on the platinum tray of the thermogravimetric analysis instrument TGA Q50 from TA Instruments, New Castle, DE, USA. Experiments were conducted in the temperature range from 25 to 800 °C under a nitrogen atmosphere, with a heating rate of 10 °C per minute.

### 2.6. Differential Scanning Calorimetry (DSC)-

Approximately 3–6 mg of each film of SR, SR-g-EGDEA, and EGDEA homopolymer were dried for 24 h at 60 °C. Runs were recorded from 25 to 400 °C at a heating rate of 10 °C per minute under a nitrogen atmosphere using a DSC 2010 calorimeter (TA Instruments, New Castle, DE, USA).

### 2.7. Swelling

Previously dried films (60 °C for 72 h), with their initial weight recorded, were immersed in a buffer solution of pH 7.4 at 37 °C. Then, films were separated from the medium, excess water was carefully removed with paper, and they were reweighed. The swelling index was calculated using the following Equation (2). Each measurement was performed in triplicate.

(2) $$Swelling\;\left(\%\right)=\frac{Wt-Wo}{Wo}$$

where Wt = weight of the film at time t and Wo = initial weight of the film.

### 2.8. Contact Angle

The contact angle was measured in triplicate at room temperature using a KRÜSS DSA 100 device (KRÜSS, Matthews, NC, USA), by placing a drop of distilled water on the surface of different films. The contact angle was recorded at 0, 5, 10, and 15 min. Each measurement was performed in triplicate.

### 2.9. Mechanical Test

Films of 1 × 3 cm were dried at 60 °C for 168 h before the test. Tensile test was conducted using a Shimadzu Precision Universal/Tensile Tester from Long Beach, CA, USA (Shimadzu Corporation) with a constant elongation rate of 0.5 mm/min.

### 2.10. Synthesis and Loading of Antimicrobial Silver

Antimicrobial silver was prepared using previously reported methods [17,18]. 0.1 g of PVP was dissolved in 20 mL of distilled water. In addition, 20 mL of an AgNO_3_ solution (10 mM) was prepared. Then, both solutions were mixed in a 1:1 ratio for 20 min. Next, the resulting solution was transferred into test tubes and exposed to γ-rays from a 60Co source (Gammabeam 651 PT, MDSNordion, Nordion (Canada) Inc., Ottawa, ON, Canada) at a dose of 20 kGy. Afterward, SR-g-EGDEA samples, 8 mm in diameter, were immersed in the antimicrobial silver solutions for 24 h. The samples were then removed and dried at room temperature for microbiological analysis.

### 2.11. Microbiological Trials

The antibacterial activity of the films was evaluated against *S. aureus* (ATCC 25923) and *E. coli* (ATCC 25922) strains with the Kirby-Bauer method. The process began by preparing Hinton Müeller agar, adjusting the pH to 7.0, and sterilizing it in an autoclave at 121 °C, 15 lb, for 15 min. The medium was then cooled to 45 °C and poured into Petri dishes to solidify. Next, the dishes were placed in an incubator at 35 °C for 24 h. The two bacterial strains were revived from ultra-freezing in their respective broths. The bacterial concentrations were adjusted to 0.5 MacFarland (MF). Starting from the 0.5 MF solution, six serial dilutions were prepared, and 100 µL of the last dilutions (10^−4^, 10^−5^, and 10^−6^) were placed on the agar. The inoculum was spread with sterile glass beads and incubated again at 35 °C for 24 h. After incubation, colony counts were performed, and calculations were made to determine the concentrations, which were 1.30 × 10^8^ for *E. coli* and 2.5 × 10^8^ for *S. aureus*. A swab from the standardized bacterial tubes was used to inoculate the surface of the culture medium in Petri dishes; samples were placed in triplicate, and the dishes were incubated at 35 °C for 24 h. After incubation, inhibition halos were measured.

## 3. Results

### 3.1. Grafting of SR-g-EGDEA by the Direct Method

The results of the degree grafting of SR-g-EGDEA are shown in Table 1. It is observed that, particularly using hexanes, there is no significant increase in grafting as previously reported [19]. This is due to homopolymerization in the EGDEA medium, which acts as a barrier to prevent gamma radiation from penetrating deeper into the SR films [20]

In terms of scalability, the direct irradiation method offers advantages over the oxidative pre-irradiation method, including shorter process times, sterilization, and control over grafting degree by adjusting the dose. Due to the type of irradiator, the ability to scale at higher levels would increase its efficiency and reduce costs [21]

### 3.2. Grafting of SR-g-EGDEA by the Oxidative Pre-Irradiation Method

As shown in Figure 1, this method involves pre-irradiating SR films with oxygen, which generates peroxides and hyperoxides in the film. These peroxides then react with the EGDEA monomer. Initially, the same solvents, petroleum ether and hexanes, were tested at 60 °C for 72 h; however, under these conditions, the grafting degree was very low (<1%). Because of this, an EGDEA solution with hexanes at 80 °C was examined. Using this method, the grafting degree ranged from 3 to 5% at different doses (10, 30, and 50 kGy), with no significant differences among them (Table 2). This is attributed to the low formation of homopolymer by this method [20]. Additionally, with this approach, grafting occurs only on the surface of SR films, unlike the direct method, where the penetrating power of ionizing gamma radiation allows it to reach deeper sites and generate random active centers along the polymer structure [22].

### 3.3. FTIR-ATR

**Figure 2:**
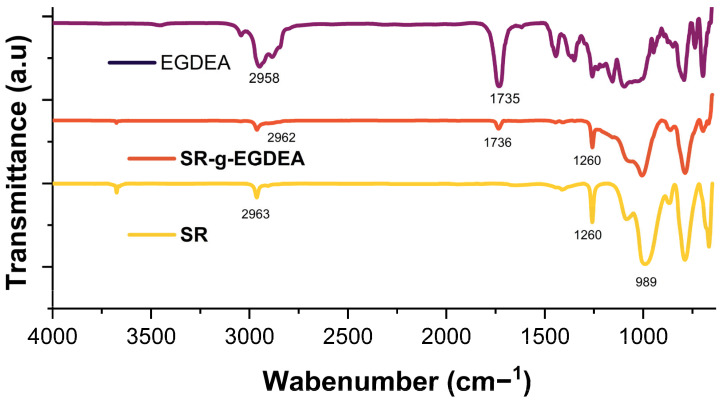
Graphs of FTIR-ATR for EGDEA homopolymer, SR, and SR-g-EGDEA film.

The FTIR-ATR spectra for the different samples are shown in Figure 2. The SR film displays characteristic bands, including a stretching band at 2963 cm^−1^ associated with methyl groups, with a confirmation band at 1260 cm^−1^, and a stretching band at 989 cm^−1^ representing the Si-O bond [23]. The modified SR-g-EGDEA film shows a band at 1736 cm^−1^ attributed to the C=O of EGDEA. Both methods reveal characteristic SR bands, demonstrating that the original properties of the SR film remain intact. The EGDEA homopolymer exhibits bands at 2958 and 1735 cm^−1^, corresponding to methyl groups and C=O, respectively [19].

### 3.4. Thermal Analysis

**Figure 3:**
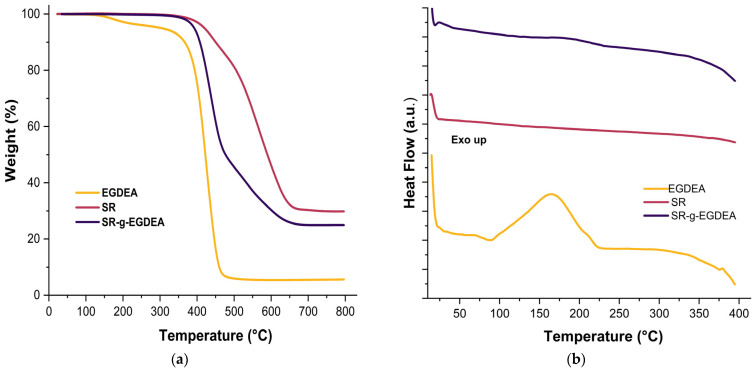
(**a**) Graphs of TGA of EGDEA homopolymer, SR, and SR-g-EGDEA film. (**b**). Graphs of DSC of EGDEA homopolymer, SR, and SR-g-EGDEA film.

Thermal analyses were conducted using TGA and DSC, as shown in Figure 3a and 3b, respectively. TGA revealed initial weight losses of 10% at approximately 450, 407, and 366 °C for SR, SR-g-EGDEA, and EGDEA homopolymer, respectively. For the SR film, only one decomposition temperature was observed, reaching up to 566 °C, with a char yield of 29.85% at 800 °C. The SR-g-EGDEA film exhibited decomposition temperatures at 438 and 586 °C, corresponding to the decomposition of EGDEA and SR, respectively, with a final char yield of 24.9% at 800 °C. Lastly, the EGDEA homopolymer showed the lowest decomposition temperature at 423.7 °C and a char yield of 5.5% at 800 °C.

DSC analysis. The SR film under the conditions studied showed no change. EGDEA homopolymer exhibits an exothermic peak at ~166 °C, which can be attributed to the decomposition of low molecular weight polymer chains formed during homopolymerization [24]. Finally, the SR-g-EGDEA film shows a slight change around ~195 °C, attributed to the decomposition of low molecular weight chains of EGDEA; the change in decomposition temperatures can be matched to the interaction of bonds between silicone and EGDEA.

### 3.5. Swelling and Contact Angle

Table 3 illustrates the swelling behavior of the SR and SR-g-EGDEA films. The SR film shows no change due to its lipophilic structure. In contrast, the SR-g-EGDEA reaches a maximum swelling of up to 8% at 60 min for both graft percentages (62.2 and 46.7%). This increase in swelling suggests that the EGDEA has reduced the lipophilicity of the SR, making it slightly more hydrophilic. The presence of the carbonyl group in the EGDEA mainly causes this.

**Figure 4:**
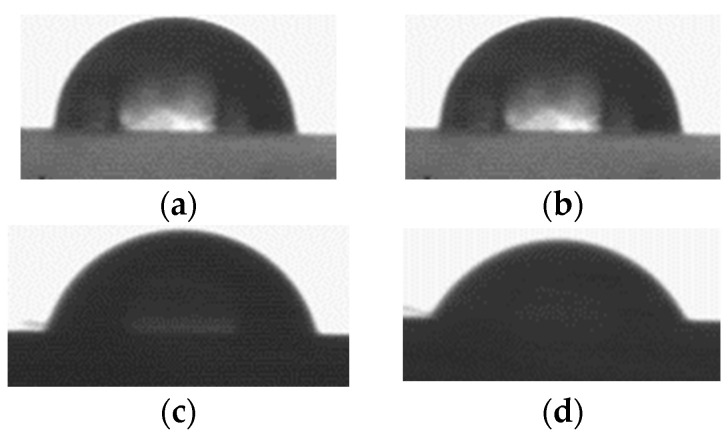
(**a**,**b**) pristine SR film at times 0 min and 15 min, respectively. (**c**,**d**) SR-g-EGDEA films with a grafting of 57.6% at times 0 min and 15 min, respectively.

Regarding the contact angle, it exhibited the same behavior. The SR film maintained a consistent contact angle of 95° ± 1 after 15 min (Figure 4a,b), similar to previously reported values of 90° [25]. For SR-g-EGDEA, the film with 3.7% grafting showed an angle of 88° ± 2, while the film with 57.6% grafting had an angle of 85° ± 4 after 15 min (Figure 4c,d). This indicates that EGDEA reduces the lipophilicity of SR films.

### 3.6. Synthesis of Antimicrobial Silver

**Figure 5:**
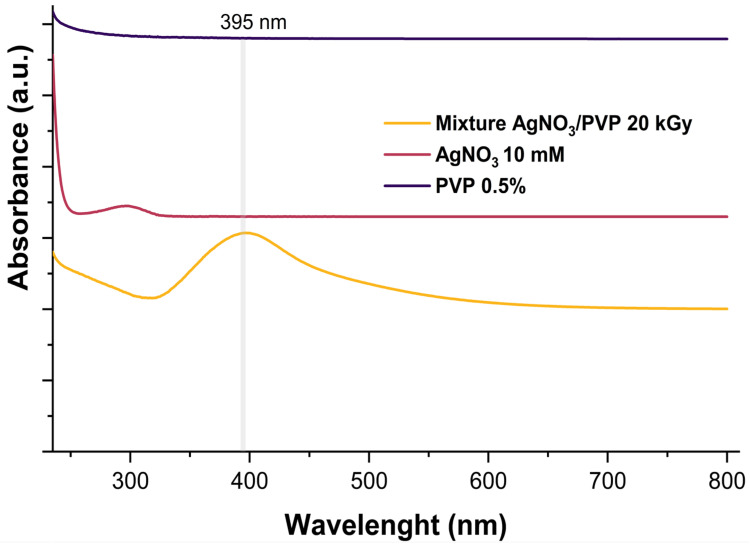
UV spectra of antimicrobial silver obtained by γ-ray irradiation reduction of silver nitrate.

The antimicrobial silver particles were synthesized by reducing silver nitrate in PVP as a stabilizer through gamma ray irradiation. After treatment, the solution turned brown, indicating the presence of silver particles and silver oxide [26,27]. UV-Vis spectroscopy analysis, shown in Figure 5, revealed a maximum absorption band at 395 nm, which literature suggests indicates the formation of AgNPs, with reported bands at 400 nm [27].

### 3.7. Antimicrobial Studies

The results of the antimicrobial test are presented in Table 4. SR-g-EGDEA control films did not show any inhibition against *E. coli* and *S. aureus*, indicating that EGDEA itself, grafted, does not have antibacterial properties. These results differ from those previously reported [10,11,12], where EGDEA is combined with other monomers, which does enhance the antibacterial property. However, it is also ascribed that the double bond involved in the polymerization of EGDEA can impact the antibacterial property of the monomer, so during in situ polymerization, this property is affected.

**Figure 6:**
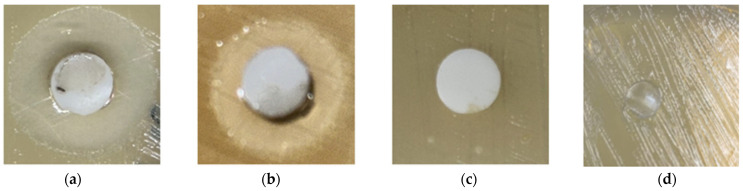
(**a**) SR-g-EGDEA loaded with antimicrobial silver tested against *S. aureus.* (**b**) SR-g-EGDEA loaded with antimicrobial silver tested against *E. coli.* (**c**) Control film SR-g-EGDEA tested against *S. aureus*. (**d**) Pristine control film tested against *E. coli*.

On the other hand, the addition of antimicrobial silver resulted in growth inhibition of both strains tested, as seen in Figure 6. The load of antimicrobial silver in SR-g-EGDEA is due to the interaction between silver ions and the carbonyl group of EGDEA [28,29]. The proposed mechanism of action is the generation of ROS (Reactive Oxygen Species), which causes cellular stress and leads to bacterial death [30]. However, the exact mechanism remains unclear [31]. The results in Table 4 match the values from 10 to 33 mm for *E. coli* and 12–35 mm for *S. aureus* [32]. Where the difference in zone of inhibition depends on the quantity of silver loaded, the size of silver particles, and the strains used [33].

### 3.8. Results of Mechanical Test

**Figure 7:**
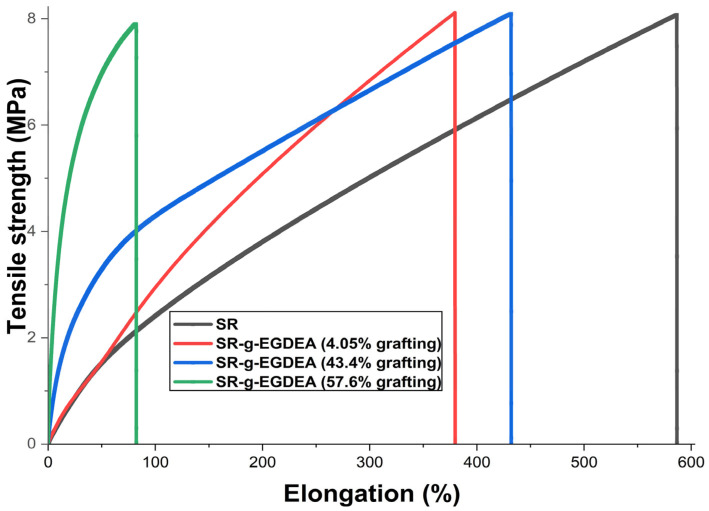
Plots of stress deformation for SR and SR-g-EGDEA films.

Figure 7 shows the graphs of the mechanical test. SR film had the highest percentage elongation and the lowest Young’s modulus, indicating that this material is more malleable (less rigid) [34] compared to its counterparts modified with EGDEA. In this context, as shown in Table 5, adding EGDEA significantly decreases the elongation of SR-g-EGDEA films. For the 57.6 and 43.4% grafts, it is attributed that a mass graft occurs, which exposes SR chains mixed with monomeric chains on the surface, reducing the malleability (elongation) of the films [35]. However, this needs further confirmation through additional characterization, such as scanning electron microscopy. Specifically, with the 57.6% graft, the material was much more rigid, which, for potential biomedical applications, would be more appropriate to apply in systems that require a rigid structural support and support high loads without suffering deformation.

## 4. Discussion

The modification of SR with EGDEA was performed using gamma ionizing radiation through both the direct method and the oxidative pre-irradiation method, with the direct method being more effective. These methods enabled the production of different films with varying grafting percentages by adjusting the radiation dose, solvents, and bath water times. Physicochemical characterizations such as DSC, TGA, FTIR-ATR, contact angle measurements, swelling, and mechanical tests confirmed the modification of SR films. SR-g-EGDEA films allowed for the loading of silver, which inhibited the growth of *E. coli* and *S. aureus* strains. These silver-loaded SR-g-EGDEA films add to the list of new materials with potential biomedical applications. Although the results are promising, further research is necessary. The grafting of other monomers can enhance the load of silver, being more effective against strains used. Techniques like atomic force microscopy (AFM) and scanning electron microscopy (SEM) will help better understand the physical and structural properties of the SR-g-EGDEA films.

## Figures and Tables

**Table 1:** Results of grafting of SR-g-EGDEA films. Direct method.

Dose (kGy)	EGDEA 25%(*v*/*v*)	Grafting (%) ^(a)^
10	Hexanes	43.6 ± 3.6
10	Petroleum ether	57.2 ± 4.5
30	Hexanes	60.1 ± 6.0
30	Petroleum ether	59.6 ± 4.5
50	Hexanes	61.6 ± 2.6
50	Petroleum ether	67.9 ± 5.5

^(a)^ Values are given as an average of five samples. (±Standard deviation).

**Table 2:** Results of grafting of SR-g-EGDEA films. Oxidative pre-irradiation method.

Dose (kGy)	EGDEA 25% (*v*/*v*)	Grafting (%) ^(a)^	Temperature (°C)	Time (hr)
10	Hexanes	4.58 ± 1.1	80	72
30	Hexanes	4.98 ± 2.1	80	72
50	Hexanes	4.51 ± 1.3	80	72

^(a)^ Values are given as an average of 5 samples. (±Standard deviation).

**Table 3:** Results of swelling of SR and SR-g-EGDEA.

Film	Time (30 min) Swelling (%)	Time (60 min) Swelling (%)
Pristine SR	-	-
SR-g-EGDEA (46.7%)	5.32	8.66
SR-g-EGDEA (62.2%)	5.77	8.44

**Table 4:** Antimicrobial results of SR-g-EGDEA loaded with antimicrobial silver against *E. coli. S. aureus*.

*E. coli*	*S. aureus*
Clear zone (mm)	Clear zone (mm)
10.06 ± 0.04	0.9 ± 0.081
11.50 ± 0.06	11.3 ± 0.28

Values are given as an average of three samples (±standard deviation). Samples possessed 64.7% of grafting.

**Table 5:** Mechanical properties for SR and SR-g-EGDEA films.

Film	Tensile Strength (MPa)	Elongation at Break (%)	Young Modulus (MPa)
SR	7.6 ± 0.47	566 ± 19.5	1.3 ± 0.07
SR-g-EGDEA (4%)	11.9 ± 0.81	324 ± 55	1.84 ± 0.06
SR-g-EGDEA (43.4%)	6.7 ± 0.89	417.5 ± 13.5	1.6 ± 0.16
SR-g-EGDEA (57.6%)	4.6% ± 0.16	72 ± 9	12.9 ± 0.26

## Data Availability

The original contributions presented in this study are included in the article. Further inquiries can be directed to the corresponding authors.
